# Cryolipolysis on More than One Body Area Increases Lipid Peroxidation without Changing Lipid Profile and Inflammatory Markers

**DOI:** 10.3390/biology11121690

**Published:** 2022-11-23

**Authors:** Antônio Daniel Saraiva da Costa, Amanda Suellenn da Silva Santos Oliveira, Ana Karolinne da Silva Brito, Lays Arnaud Rosal Lopes, Maísa Guimarães Silva Primo, Ana Lina de Carvalho Cunha Sales, Marcos Antônio Pereira dos Santos, Nara Vanessa dos Anjos Barros, Oséas Florêncio de Moura-Filho, Jaynara Keylla Moreira da Silva, Edwiges Ita de Miranda Moura, Massimo Lucarini, Alessandra Durazzo, Daniel Dias Rufino Arcanjo, Maria do Carmo de Carvalho e Martins

**Affiliations:** 1 Postgraduate Program in Food and Nutrition, Federal University of Piauí, Teresina 64049-550, Brazil; 2 Department of Biophysics and Physiology, Federal University of Piauí, Teresina 64049-550, Brazil; 3 Campus Helvídio Nunes de Barros (CSHNB), Federal University of Piauí, Picos 64607-670, Brazil; 4 Institute of Teaching and Research in Clinical Physiotherapy, Teresina 64049-550, Brazil; 5 MedImagem Group, GMI, Teresina 64049-550, Brazil; 6 CREA-Research Centre for Food and Nutrition, Via Ardeatina 546, 00178 Rome, Italy

**Keywords:** cryolipolysis, oxidative stress, inflammation, body composition, subcutaneous fat, adipose tissue

## Abstract

**Simple Summary:**

Cryolipolysis is a technique based on reduction of skin temperature, leading to local or systemic exposure of subcutaneous adipose tissue to active cooling, which are able to cause possible inflammatory and oxidative stress repercussions. In the present study, cyolipolysis in adult women did not change body composition, lipid profile or inflammatory markers; when applied on the abdomen and flanks, this procedure led to an isolated increase in lipid peroxidation markers. Besides no cardiometabolic risk using this procedure was observed.

**Abstract:**

In the present study, the effects of cryolipolysis on one and multiple body areas, assessing body composition, lipid profile and peroxidation and inflammatory markers were investigated. Twenty-four women aged between 20 and 59 years were randomly assigned to three groups: (1) control, (2) cryolipolysis on the abdomen and (3) cryolipolysis on the abdomen + flanks. Anthropometric measurements, bioimpedance and ultrasound were performed, as well serum lipid profile, lipid peroxidation markers (malondialdehyde and myeloperoxidase) and inflammatory markers (C-reactive protein and Interleukin-1β) were determined. In addition, food consumption and physical activity level were evaluated. Data were obtained at 0, 10 and 30 days (t0, t10 and t30) after cryolipolysis. Cryolipolysis did not change anthropometric measurements, body composition or lipid profile. Interestingly, the abdomen + flanks group had significantly increased plasma myeloperoxidase activity at t0, t10 and t30, and increased malondialdehyde levels at t0 and t10 when compared to the other groups. Furthermore, there were no differences between macronutrient intake and total energy value, physical activity level, malondialdehyde and interleukin-1β at t30. Cryolipolysis did not change body composition, lipid profile or inflammatory markers investigated. On the other hand, when used on the abdomen and flanks, it produced an increase in lipid peroxidation markers, malondialdehyde and myeloperoxidase.

## 1. Introduction

Adipose tissue is an organ with multiple functions, and excessive adiposity plays a central role in the pathogenesis of several chronic non-communicable diseases (NCDs) [1,2]. Adipocyte activity is modulated by the action of hormones, such as insulin, cortisol and catecholamines; in response, a variety of substances which act locally and/or systemically are secreted, and then participate in the regulation of endothelial function and energy balance, as well as in the sensitivity to insulin action and the process of atherogenesis [1,2,3,4]. The metabolic role of adipocytes can be influenced by their location and fat content, with intra-abdominal adiposity having the highest impact on metabolism, including changes in the sensitivity to insulin action [2,5].

In this context, overweight and obesity with central body fat deposition are associated with increased cardiovascular risk and several NCDs [6,7]. Dietary interventions for the treatment of overweight and obesity bring benefits that can be observed in the short term. Some studies have sought alternative strategies for the ablation of adipose tissue, including its surgical removal and the use of radiofrequency, ultrasound and laser [8,9]. Another alternative consists of cooling the body by environmental exposure to cold [10,11,12,13] or through devices to reduce skin temperature, leading to local or systemic exposure of subcutaneous adipose tissue to active cooling, a technique called cryolipolysis.

The conception of the idea of applying cryolipolysis in the treatment of localized fat is based on the observations of Epstein e Oren [14], who proposed the term popsicle panniculitis for the presence of a red indurated nodule and a transient fatty necrosis on the cheek of a child associated with popsicle intake. So, in 2007, Manstein et al. [15] presented a new non-invasive method for reducing body fat through freezing, a technique called cryolipolysis.

In 2010, the Food and Drug Administration released cryolipolysis equipment for use in procedures aimed at reducing localized fat in the flanks and abdomen and in 2014, the same agency released this equipment to treat subcutaneous fat located in the thighs [16].

Cryolipolysis is a non-invasive method that acts on the subcutaneous adipose tissue by means of controlled cooling, with a temperature ranging from −5 to −15 °C, without causing damage to the underlying epidermis and dermis [17,18,19]. A hypothesis that can explain the action of cryolipolysis on fat reduction in adipocytes suggests that exposure to cold would act to increase energy expenditure through lipid oxidation and thermogenesis, resulting in a decrease in cellular fat mass in the short term, without apoptosis [20,21]. Another hypothesis is that adipose cells are more sensitive to this procedure than cells from other tissues and, thus, cooling could lead to crystallization of cytoplasmic lipids, loss of cellular integrity, apoptosis/necrosis of adipocytes and local inflammation, producing selective loss of adipose tissue, over weeks to months, with a non-immediate response [22,23,24,25].

The effects of cryolipolysis have been the object of studies that have sought to relate the use of the method to changes in weight and body composition [20,24,25,26,27,28,29,30]. There is also interest in the destination of the fat released after apoptosis of adipocytes, as well as in the magnitude of the reactions induced by the procedure and its impact on systemic homeostasis, especially on serum lipid concentrations, liver function, inflammatory state and oxidative stress [31]. Regarding the destination of fat resulting from apoptosis, there is still no conclusive study on this aspect, but it has been suggested that it would be eliminated after its release, from adipocytes, into the bloodstream [20].

Moreover, upon the completion of cryolipolysis and completion of the vacuum, blood reperfusion occurs in adipocytes, and the reoxygenation of the treatment areas could induce an inflammatory state, which would be initiated by the generation of reactive oxygen species, activation of proteolytic enzymes (caspases), which would potentiate adipocyte cell death [22]. Recently, a split-body in women trial showed how a single unilateral session of noninvasive selective cryolipolysis can be considered as a safe and effective treatment for reduction in visceral adipose tissue over a period of 12 weeks, which should result in metabolic improvement [32]. It is worth mentioning the recent study of Abdel-Aal et al. [33] that, by investigating the systemic effects of cryolipolysis in central obese women in a randomized controlled trial showed how women who underwent cryolipolysis and under a dietary program had better improvement in components of lipid profile and liver enzymes than women who were maintained over diet program alone: measures to decrease waist circumference, associated with abdominal subcutaneous fat reduction, enhanced the systemic effects of cryolipolysis.

In view of the above, the relevance of characterizing the possible inflammatory and oxidative stress repercussions induced by cryolipolysis in the short term is evidenced, since the increase in oxidative stress contributes to the pathogenesis of cardiovascular diseases and has feedback mechanisms related to changes in the inflammatory state [26,33].

Thus, in this context, the aim of this research which investigates the effects of cryolipolysis on systemic homeostasis is justified, contributing with information about the potential risks and benefits of the use of cryolipolysis, and the magnitude of systemic effects of its use in relation to the number of areas subjected to the procedure. Therefore, this study evaluated the effects of cryolipolysis used on one and multiple areas of the body, on body composition, on lipid profile, and on inflammatory and peroxidation markers in women.

## 2. Materials and Methods

### 2.1. Participants

This is an open clinical trial with parallel groups from a convenience sample including 24 adult women living in Teresina, Piauí. Recruitment took place between December 2019 and January 2020. Inclusion criteria were as follows: age between 20 and 59 years; accumulation of localized fat in the abdomen and flanks and abdominal circumference higher than or equal to 80 cm.

Exclusion criteria were: presence of obesity, hernias (umbilical, inguinal), varicose veins or inflammatory and/or infectious lesions on the skin of the area of the body where cryolipolysis was used; women in the gestation or lactation period; previous diagnosis of diabetes mellitus, systemic lupus erythematosus, rheumatoid arthritis, Sjögren’s syndrome or cryoglobulinemia; paroxysmal cold hemoglobinuria (autoimmune hemolytic anemia); surgical procedure previous in the body area where cryolipolysis was used; presence of vasospastic phenomena (Raynaud’s syndrome and acrocyanosis); anticoagulants use; presence of neurological disease; treatment with dietary restriction/dietary re-education; steroidal or non-steroidal anti-inflammatory drugs use; active inflammatory disease; smoker.

**Figure 1:**
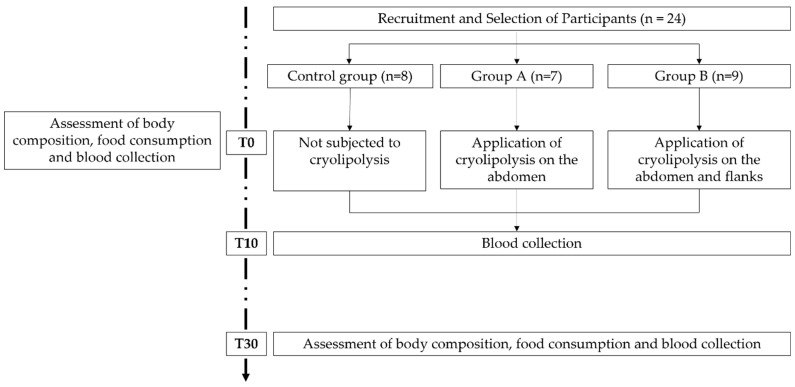
Study design.

The selected participants were randomly distributed into three groups: group A: Women subjected to the application of cryolipolysis on the abdomen (*n* = 7); group B: Women subjected to the application of cryolipolysis on the abdomen and flanks (*n* = 9); and Control group: women not subjected to cryolipolysis (*n* = 8). After allocation, procedures were scheduled. All data described below were collected at times 0 (t0), 10 (t10) and 30 (t30) days after application of cryolipolysis (Figure 1).

### 2.2. Anthropometric Assessment

Body weight, height, waist circumference (WC), hip circumference (HC) and abdomen circumference (AC) measurements were taken. Body weight and height were measured in triplicate to obtain the mean of the measurements [34] by a digital scale (Líder^®^, model P150C, capacity for 200 kg, graduated in 100 g, Araçatuba, SP, Brazil). From these measurements, the Body Mass Index (BMI) was calculated from the average value of body weight (kg) divided by the average value of height (m) to the power of 2, aiming to classify the global nutritional status, adopting the criterion defined for adults by the World Health Organization [35].

Circumference measurements were performed using a non-extensible Seca^®^ measuring tape (Hamburg, Germany), with the individual in an upright position. WC was measured with the tape encircling the waist one centimeter above the height of the umbilicus [36]. AC was obtained by placing the tape at the level of the umbilical scar [37]. HC was measured encircling the widest region of the hip [38].

### 2.3. Body Composition

Body composition was determined by means of bioelectrical impedance (BIA) using a Biodynamics^®^ tetrapolar electrical bioimpedance analyzer (Shoreline, WA, USA), model 310, version 8.01. The evaluation was performed according to the procedures described by Cômodo et al. [39].

Ultrasonography (US) images were also obtained for the determination of subcutaneous fat using a Bodymetrix^®^ portable ultrasound device (Araraquara, São Paulo, Brazil) connected to a computer with BodyView^®^ software (version 7.0) [40]. The machine showed that the interfaces between the fat–muscle and muscle–bone body layers had different reflection fractions the (R = 0.012 and R = 0.22, respectively), allowing the dimensioning of these layers. Measurements were taken in the following areas of the body: Chest, Triceps, Subscapularis, Middle Axillary, Suprailiac, Abdomen, and Thigh. Fat percentage was determined using the Jackson and Pollok [41] equation, and this percentage was calculated using the equipment’s software.

### 2.4. Physical Activity Level

To determine Physical Activity Level (PAL), the International Physical Activity Questionnaire (IPAQ) classification criteria [42] was used, with participants classified as active, irregularly active and sedentary.

### 2.5. Food Consumption

Food consumption was assessed using a 24-h dietary recall questionnaire. The amounts of food consumed, expressed in home measurements, were converted into grams or milliliters with the help of a Photographic Manual for Dietary Surveys and Food Composition Tables [43], with the aim of standardizing information. The match of national Food Composition Databases, including data on sample foods, processed foods and complex food matrices, with food consumption data, are essential Key tools in nutrition and health area [44].

Subsequently, the foods were broken down into ingredients to calculate the nutritional value of macronutrients (carbohydrates, proteins, lipids) and the total energy value (TEV), using the software AvaNutri Online^®^ version 4.0 Revolution (AvaNutri e Nutrição, Três Rios, RJ, Brazil (Available in https://www.avanutri.com.br/software_avanutri_online/. Accessed on 15 September 2022 [45], utilizing food chemical composition tables [46,47].

### 2.6. Lipid Profile

Serum concentrations of triglycerides, total cholesterol and high-density lipoprotein-bound cholesterol (HDL-cholesterol) were determined using the dry chemistry method. The LDL-cholesterol fraction was calculated according to the formula by Friedwald et al. [48]. Blood samples were obtained after a 12-h fast.

### 2.7. Malondialdehyde Concentration and Myeloperoxidase Activity in Plasma

Malondialdehyde (MDA) concentrations were determined by measuring the production in thiobarbituric acid reactive substances (TBARS) [49]. A mixture of 200 µL of plasma with 350 µL of 20% acetic acid (pH 3.5) and 600 µL of 0.5% TBARS was incubated in a water bath for 45 min at 100 °C. After cooling in an ice bath for 15 min, 50 µL of 8.1% sodium dodecyl sulfate was added. The mixture was centrifuged for 15 min at 12,000 rpm at 25 °C. The supernatant was read in a spectrophotometer at wavelengths of 532, 510 and 560 nm for absorbance correction using the Pyles formula [50]. An analytical calibration curve was prepared using MDA as a standard. Results were expressed as nmol MDA per mL of plasma.

The measurement of myeloperoxidase (MPO) activity was based on the rate of oxidation of the substrate o-dianisidine in the presence of H_2_O_2_ and evidenced by the change in absorbance measured at 450 nm [51]. The reading was performed in an ELISA microplate with 10 µL of plasma and 200 µL of the reading solution, composed of distilled H_2_O, phosphate buffer pH 6.0, 1% H_2_O_2_ and 5 mg of o-dianisidine. Two readings were taken at 450 nm absorbance at 1-min intervals. Results were expressed as units of MPO/L of sample (U/L).

### 2.8. Serum Concentration of Interleukin-1β and C-Reactive Protein

To assess inflammatory state, serum concentrations of the markers of Interleukin-1β (IL-1β) and C-Reactive Protein (CRP) were quantified. The determination of the concentration of CRP was performed by heterogeneous enzyme sandwich immunoassay, using CRP Slides from Vitros^®^ Chemistry Systems (Raritan, Somerset, NJ, USA), version 4.0. Values > 1.0 mg/dL were considered indicative of the presence of inflammatory processes, as specified in the manufacturer’s manual for the equipment used in the analysis. The determination of plasma concentration of IL-1β was performed using an ELISA kit (ThermoFisher^®^, São Paulo, Brazil). The analysis was performed according to the manufacturer’s recommendations. Results were expressed in ng/L.

### 2.9. Application of Cryolipolysis

The cryolipolysis machine used was Polarys^®^, manufactured by Imbramed^®^, with application time used commercially in a single 60-min session at CIF 42 (10 °C). A large size vacuum applicator (20 cm wide) was used for the abdomen and medium size applicators (15 cm wide) for the flanks. Two application units were used, both with the same control system, allowing practically the same cooling capacity. The time between the application of cryolipolysis in the abdomen region to the flank regions, in group B, was less than 30 min. Subsequently, a clinical evaluation of the treatment areas was performed to identify signs of possible adverse reactions to the procedure. Group C was not subjected to cryolipolysis.

### 2.10. Data Analysis

Data distribution was tested using the Kolmogorov–Smirnov test. Normally distributed variables were presented as mean and standard deviation and the comparison between groups or times of application of cryolipolysis was performed using Student’s *t*-test or One Way ANOVA followed by Student–Newman–Keuls post-test. Variables with non-normal distribution were expressed as median and interquartile range, and the comparisons were tested using the non-parametric Mann–Whitney or Kruskal–Wallis tests and Dunn’s post-test, adjusted by the Bonferroni method. A 5% significance level and a 95% confidence interval were adopted. Analyzes were performed using Stata 14.0 program.

### 2.11. Ethical Aspects

The research project was approved by the Comitê de Ética e Pesquisa da Universidade Federal do Piauí (Ethics Committee on Research of Federal University of Piauí), *parecer* (opinion) No. 3,429,588. All procedures were performed in accordance with the Declaration of Helsinki. The participants received clarification about the study and confirmed their participation in the study by signing an informed consent form.

## 3. Results

The sample consisted of 24 women, where 7 are from control group, 8 are from group A and 9 are from group B. It was not found statistically significant difference (*p* > 0.05) regarding the age of the participants in the studied groups (control group: 32.3 ± 10.5 years; group A: 32.1 ± 8.4 years; and group B: 35.9 ± 7.5 years). There were no reports or signs of adverse reactions resulting from the application of cryolipolysis. Table 1 presents the anthropometric and body composition characteristics of the study participants. No statistically significant differences were found between the beginning (t0) and the end of 30 days of treatment for each group (t30) or between the experimental groups regarding the variables analyzed in Table 1.

There were also no statistically significant differences regarding food consumption (Table 2) and PAL (Table 3) between the studied groups and at the end of the follow-up time after cryolipolysis.

Although no significant difference was found in the components of the lipid profile between the groups (Table 4), it was found that the participants, but t0 in group B, had mean HDLc concentrations below the desirable levels at times t0 and t30.

When analyzing the inflammation markers, it was observed that approximately half of the participants of the three experimental groups had CRP concentrations considered as indicative of the presence of inflammatory processes (CRP > 1 mg/dL) (Table 5). On the other hand, for IL-1β concentrations (Table 6), no statistically significant differences were found between the means of the evaluated groups.

Regarding lipid peroxidation markers (Table 6), the mean MDA concentration was significantly higher in group B (cryolipolysis on the abdomen and flanks) when compared to the other groups at times t0 and t10, but not at t30. In the control group, the mean values obtained at t30 were significantly higher than those found at t0 and t10, and in group A, the mean values at times t10 and t30 were significantly higher than at t0.

As for the concentrations of MPO, the values were significantly higher in group B (cryo-lipolysis on the abdomen and flanks) at times t0 and t10 when compared with the other groups at the corresponding time. At t30, group B had significantly higher concentrations compared to the Control group, but not in relation to group A (cryolipolysis on the abdomen).

## 4. Discussion

The relevance of this study is based on a low metabolic risk resulting from the application of cryolipolysis on the abdomen or abdomen and flanks for up to 30 days, considering that no changes were found in global nutritional status, body composition, lipid profile and systemic inflammation markers analyzed, despite having resulted in an increase in MDA concentration and MPO activity in plasma.

Cryolipolysis did not produce body weight loss or BMI reduction, corroborating the results of other studies [20,24,25,26,27], in which body weight remained unchanged soon after and up to 90 days after the application of cryolipolysis on the abdomen or flanks, without simultaneous applications. A possible explanation for the absence of changes in these parameters could be related to the fact that cryolipolysis is not aimed at weight loss, but to promote the reduction in subcutaneous localized fat [18].

In this investigation, weight was measured between 0 and 30 days, a short period of time compared to that generally described to obtain the effects of the cryolipolysis on the reduction in body measurements. The choice of a 30-day period was based on the hypothesis proposed by Loap and Lathe [52] that the reduction in adipose tissue through cryolipolysis can be mediated by thermogenic fat metabolism without adipocyte apoptosis. However, the results of this study, regarding weight, WC and BMI, were different from those described by Loap and Lathe [52] using a different protocol, which consisted of performing six daily sessions of cryolipolysis.

In this study, there were not found effects of cryolipolysis on body composition determined by BIA and US. These results are in agreement with those found by Falster et al. [53], in which there were no significant differences between the intervention and control groups regarding the thickness of the adipose layer evaluated by US at any of the times evaluated by them (t0, t30, t60 and t90). Probably, the absence of alterations is partly justified by the fact that the effects of cryolipolysis are restricted to the application areas, in which the minimum period of three months is described as the necessary time for the manifestation of the final result, with little or no change in weight and body composition [20,27].

In this sense, in a study developed by Ponga-Manso [54] it was observed that six months after the application of cryolipolysis there was a reduction in the thickness of the abdominal fat layer. Also in the research carried out by Savacini et al. [55] statistically significant reductions in the fat layer in the abdomen and flanks were demonstrated at the evaluated times (t0, t30, t60 and t90). Furthermore, BIA is an indirect method of assessing body composition, with body compartments being estimated by means of statistical derivation from comparison with gold standard methods. Thus, small changes may not be detected by BIA due to its low sensitivity [51].

By analyzing possible interference of food consumption and physical activity level (PAL) of the participants in the results of global nutritional status and body composition, no increases were found in macronutrient intake in the total energy value. Similarly, no significant change in PAL at the end of the 30-day period was observed, despite a trend towards an increase in the PAL of the participants. In this study, although one of the groups was subjected to cryolipolysis on three areas (group B), which could cause higher acute inflammatory changes with subsequent adipocyte death, which could induce changes in the lipid profile, no changes in the lipidogram were found. Similarly, Loap and Lathe [52], when submitting women to cryolipolysis in the lower regions of the back and abdomen, as well as the hip, also did not identify acute changes in the lipid profile, although the authors performed the evaluation after only 3 days with 6 daily cryolipolysis sessions.

In a study carried out by Savacini et al. [55] with 21 men and women aged between 18 and 50 years, treated with contrast cryolipolysis in the abdominal regions and/or flanks, no significant changes were found in the lipid profile after three weeks of application of cryolipolysis in the regions of the abdomen and flanks. An aspect mentioned in the study by Klein et al. [56] is that fat reabsorption after cryolipolysis can occur at a slow pace, from 60 to 120 days on average, without systemic repercussions, since adipocyte cell death would not happen simultaneously, as it was demonstrated by histological analysis of adipose tissue before and after application of cryolipolysis, for a period of 120 days after intervention. Thus, cryolipolysis seems to be a procedure without significant repercussions on the lipid profile within 30 days after application, since this procedure did not have negative impacts on the lipid profile, that is, it did not produce direct changes in the biochemical parameters of the general metabolic state analyzed here.

Likewise, no changes were observed in the inflammatory markers analyzed in this study, CRP and IL-1β. CRP plays a central role in cardiovascular disease, while IL-1β plays a key role in acute and chronic inflammatory and autoimmune diseases [57]. Absence of evidence of systemic inflammation in this study supports the hypothesis of the involvement of a possible underlying mechanism that contradicts the one that proposes the rupture of adipocytes, which could involve the reduction in adipocyte volume through thermogenesis mechanism, maintaining cell integrity [58]. Another point to be highlighted is that cryolipolysis was performed on relatively small body areas in relation to the total body surface area and, therefore, it was possibly not able to induce systemic inflammatory responses.

However, this fact does not exclude the presence of localized inflammation, as demonstrated in a study by Pugliese et al. [58], conducted in six women subjected to a single session of cryolipolysis in the abdominal region, and in which the peak of localized inflammation occurred between the 10th and 15th day. After the 30th to the 45th day, the presence of fibroblasts became more apparent, characterizing the beginning of tissue repair and the reduction in small-localized inflammation. Other studies analyzing histological aspects [15,58,59,60] showed an inflammatory process located at similar times to those described by Pugliese et al. [58].

Thus, it can be inferred that although cryolipolysis induces localized inflammation in the treatment area, it would not induce systemic inflammation, characterizing a positive aspect in the sense that it does not generate a contraindication for the use of the procedure, both for individuals with low-grade chronic inflammation and for those with active systemic inflammatory disease, such as inflammatory bowel disease or autoimmune diseases [61].

In addition, another aspect to be considered in this study is that the changes in the inflammatory profile may have been minimized due to the change in the number of physically active participants at the end of the study, which resulted in a change in energy expenditure. In this sense, Flynn, McFarlin and Markofski [62] reported that the practice of physical exercise demonstrates anti-inflammatory action, which is independent of the loss of body fat, is an effect seen both in people of any age group as well as in individuals with chronic diseases.

The anti-inflammatory effect attributed to regular physical exercise may be mediated by mechanisms that include: 1. the release of interleukin-6 into the bloodstream from contracted muscle fibers, which stimulate increased production of interleukin-10 and the interleukin-1 antagonist receptor or 2. increased concentrations of regulatory T cells that are identified as interleukin-10 secreting cells; 3. repression of Toll-like receptor gene expression in monocytes and inhibition of downstream responses; 4. decreased levels of monocytes that stimulate the inflammatory process; and 5. inhibition of the process of infiltration of monocytes and/or macrophages in adipocytes [63].

As for the lipid peroxidation markers MDA and MPO, analyzed to determine the possible presence of oxidative stress, it is noteworthy that although food consumption and PAL have not undergone significant changes, the variation in the consumption of micronutrients and other components can interfere with the antioxidant activity, and this aspect was not controlled in this research. Minerals such as zinc and selenium, for instance, play an important role in oxidative stress and inflammation [64,65], and a deficiency in these micronutrients could exacerbate these processes. In addition, overweight and obesity per se also consist in precipitating factors of oxidative stress [65].

The concentrations of MDA quantified in this research were lower than those reported in obese individuals in the study by Olusi [66], which evaluated lipid peroxidation in patients with different degrees of obesity. In addition, although cryolipolysis induced significant changes in MDA concentrations, these remained at values below those identified in people with chronic diseases, such as obesity.

MDA is an aldehyde formed at the end of the oxidation of polyunsaturated fatty acids, considered a good marker of oxidative stress [66]. MDA can react with organic compounds such as deoxyribonucleic acid (DNA) and proteins, and the reaction of two aldehyde groups with nucleophiles allows MDA to generate adducts, which produce toxic effects on biological molecules [67].

MPO is a hemeprotein used as a marker of endothelial oxidative stress: the decrease in its concentration indicates a reduction in inflammatory and oxidative processes [68]. A study by Zaki et al. [69] with obese women showed higher MPO activity compared to that found in women in the control group. The authors identified that women with the highest tertile of MPO activity had higher concentrations of the lipidogram components, in addition to increased diastolic and systolic blo od pressure and HOMA-IR than those classified in the lowest tertile.

MPO has been linked to the pathogenesis of numerous diseases, including inflammatory, neurodegenerative diseases, obesity, diabetes, cancer, and heart disease, in which chronic or acute inflammatory processes are often present [70,71]. The harmful action of MPO in cells is mediated, in general, by oxidizing agents from the enzyme itself, which react with biomolecules causing toxic effects [70].

In this study, cryolipolysis does not change/affect the concentration of MPO, by indicating how this procedure may not increase the cardiometabolic risk in people having altered plasma concentrations of MPO, such as those with heart disease.

Although the marked relevance of these findings, it is worthy to report some limitations of this study, as follows: (i) use of restricted follow-up times of up to 30 days, as the effects of cryolipolysis are considered to have a slow onset of up to 90 days; (ii) the analysis of lipid peroxidation markers only, since for a more comprehensive assessment of possible oxidative stress state it would also be interesting to evaluate markers of antioxidant activity; (iii) micronutrient intake and psychological stress condition were not evaluated, which are important confounding variables; (iv) the study reflects local data not corresponding to a global scale, so the results obtained may reflect a regional influence on the genetics of the participants; (iv) the study reflects local data, not corresponding to a global scale, so that the results obtained may reflect a regional influence on the genetics of the participants; and (v) the study did not assess liver enzymes, cortisol and other markers of metabolic pathways that could indicate where fat may have moved. (vi) the study did not assess liver enzymes, cortisol and other markers of metabolic pathways that could indicate where fat may have eliminated.

Despite these limitations, this study provides important information regarding the safety of the cryolipolysis procedure performed on the areas of the abdomen and flanks, showing that there are no negative repercussions of the application in a period of up to 30 days regarding the lipid profile and inflammatory and lipid peroxidation markers. Moreover, the evaluation of lipid peroxidation markers was an innovative aspect in this study, as no other studies that analyzed the effects of cryolipolysis on these parameters were identified.

## 5. Conclusions

Cryolipolysis, used on one or more body areas of adult women with central adiposity, did not change body composition, lipid profile and inflammatory markers. When cryolipolysis is applied on the abdomen and flanks, this procedure led to an isolated increase in lipid peroxidation markers. The analysis of the results obtained, in a period of up to 30 days, indicates that cryolipolysis did not produce changes indicative of increased cardiometabolic risk. However, the absence of changes in anthropometric and body composition measurements may be related to the short period evaluated, since the effects indicated for the reduction in subcutaneous fat promoted by cryolipolysis occur more slowly, in up to 90 days.

## Figures and Tables

**Table 1:** Anthropometric and body composition characteristics of adult women subjected to cryolipolysis on one (group A) or multiple areas of the body (group B).

	Control Group (*n* = 8) (Mean ± SD)		Group A (*n* = 7) (Mean ± SD)		Group B (*n* = 9) (Mean ± SD)	
	t0	t30	t0	t30	t0	t30
Anthropometric characteristics						
Body weight (kg)	69.4 ± 8.4	69.7 ± 8.6	67.1 ± 8.0	67.3 ± 7.7	66.8 ± 9.7	66.9 ± 9.7
Abdomen circumference (cm)	88.8 ± 8.6	90.0 ± 9.0	85.4 ± 6.7	83.9 ± 6.5	85.0 ± 7.5	85.7 ± 8.0
Waist circumference (cm)	75.7 ± 24.6	83.2 ± 8.7	76.2 ± 5.6	75.4 ± 5.3	77.7 ± 7.6	76.2 ± 8.8
Hip circumference (cm)	103.2 ± 8.0	103.8 ± 8.4	101.4 ± 5.8	101.4 ± 6.4	99.8 ± 12.1	102.4 ± 8.6
BMI (kg/m^2^)	27.2 ± 3.8	27.3 ± 3.9	25.3 ± 2.9	25.4 ± 2.7	25.6 ± 4.5	25.6 ± 4,6
Body composition components determined by bioimpedance						
Body fat (%)	32.4 ± 5.6	33.9 ± 4.4	30.3 ± 1.9	32.5 ± 2.5	29.5 ± 4.0	31.8 ± 4.7
Fat weight (kg)	22.9 ± 6.3	24.2 ± 5.9	20.4 ± 3.3	22.0 ± 4.0	19.9 ± 6.0	21.2 ± 7.2
Fat-free mass weight (kg)	42.5 ± 11.0	42.1 ± 10.7	46.6 ± 5.0	45.2 ± 4.0	46.9 ± 5.1	45.5 ± 4.1
BMR (kcal/day)	1416 ± 96.0	1388 ± 100.6	1421 ± 152.3	1375 ± 123.5	1424 ± 115.8	1383 ± 122.2
Total body water (kg)	34.7 ± 7.4	33.8 ± 8.2	32.3 ± 3.3	31.1 ± 2.5	33.7 ± 3.9	40.0 ± 17.3
Body composition components determined by ultrasound						
% Body fat (%)	33.5 ± 4.1	32.8 ± 4.0	30.8 ± 4.3	29.7 ± 5.0	30.8 ± 5.7	31.3 ± 5.7
Healthy fat weight (kg)	14.5 ± 1.0	14.7 ± 1.3	16.4 ± 7.0	16.6 ± 7.0	14.3 ± 1.6	16.5 ± 8.4
Fat-free mass (kg)	14.9 ± 7.3	26.9 ± 40.8	12.4 ± 1.1	12.6 ± 1.3	12.3 ± 1.4	12.0 ± 1.3
Total body water (kg)	33.4 ± 2.4	34.0 ± 2.9	33.5 ± 3.3	34.1 ± 3.5	30.6 ± 5.9	32.7 ± 3. 5
Abdominal fat (mm) *	31.6 ± 9.9	32.4 ± 11.6	30.1 ± 6.9	29.7 ± 6.4	27.6 ± 8.5	29.9 ± 6.6
Flank fat (mm) **	17.7 ± 3.8	16.8 ± 4.2	16.9 ± 3.0	16.8 ± 2.5	21.2 ± 10.7	17.9 ± 7.4

t0 = 0 days; t30 = 30 days; Mean ± SD (Standard Deviation). BMI (Body Mass Index); BMR (Basal Metabolic Rate) * Abdominal Subcutaneous Fat Thickness; ** Flank subcutaneous fat thickness.

**Table 2:** Food consumption of adult women subjected to cryolipolysis on one (group A) or multiple areas of the body (group B).

	Control Group (*n* = 8)		Group A (*n* = 7)		Group B (*n* = 9)	
	t0	t30	t0	t30	t0	t30
Carbohydrates (g) (Median ± IQR)	214.2 ± 176.0	210.0 ± 192.8	397.6 ± 269.1	166.4 ± 190.3	135.8 ± 47.7	170.0 ± 116.4
Proteins (g) (Median ± IQR)	93.1 ± 69.6	90.1 ± 70.0	127.2 ± 20.6	60.2 ± 68.1	82.8 ± 71.1	86.2 ± 42.7
Lipids (g) (Median ± IQR)	69.5 ± 53.4	54.9 ± 70.5	71.0 ± 65.3	46.7 ± 37.0	45.3 ± 24.7	57.6 ± 85.3
TEV (kcal) (Mean ± SD)	1256.0 ± 151.3	1547.0 ± 579.2	1626.6 ± 591.0	1307 ± 686.6	1461.0 ± 576.3	1252.8 ± 393.1

t0 = 0 days; t30 = 30 days; Mean ± SD (Standard Deviation); Median ± IQR (Interquartile Range).

**Table 3:** Irregularly active and sedentary adult women according to time after cryolipolysis on one (group A) or multiple areas of the body (group B).

	t0			t30		
	*n*	c (%)	*p*	*n*	c (%)	*p*
Control group (*n* = 8)	8	2 (25.0)	0.078	8	0 (0.0)	0.065
Group A	7	2 (28.6)		7	1 (14.3)	
Group B	9	6 (66.7)		9	3 (33.3)	

t0 = 0 days; t30 = 30 days; *n*: number of participants in the group; c: number of cases; chi-square association test by time according to group.

**Table 4:** Lipid profile of adult women subjected to cryolipolysis on one (group A) or multiple areas of the body (group B).

	Control Group (*n* = 8) (Mean ± SD)			Group A (*n* = 7) (Mean ± SD)			Group B (*n* = 9) (Mean ± SD)		
	t0	t10	t30	t0	t10	t30	t0	t10	t30
TC	126 ± 27.8	149 ± 38.9	119.5 ± 40.3	150.5 ± 13.6	131.6 ± 29.6	126.5 ± 21.4	129 ± 24.0	129.4 ± 27.9	137.3 ± 42.2
TG	91 ± 31.3	115.5 ± 70.9	98.6 ± 43.1	88.8 ± 34.5	82.1 ± 20.1	76.3 ± 31.3	116 ± 38.1	87.5 ± 43.3	100.2 ± 57.3
LDLc	72.4 ± 26.8	87.9 ± 22.3	71.2 ± 26.8	95.7 ± 14.7	82 ± 21.4	82.3 ± 13.6	90.7 ± 18.2	81.8.1 ± 13.2	80.5 ± 33.5
HDLc	35.4 ± 5.5	37.9 ± 13.4	28.1 ± 10.8	37.5 ± 8.2	33.1 ± 13.4	29.3 ± 8.2	45.2 ± 15.8	34.9 ± 11.9	36.6 ± 9.7

t0 = 0 days; t10 = 10 days; t30 = 30 days; Mean ± SD (Standard Deviation), TC (Total Cholesterol); TG (Triglycerides); LDLc (low-density lipoprotein-bound cholesterol); HDLc (high-density lipoprotein-bound cholesterol).

**Table 5:** Distribution of adult women subjected to cryolipolysis on one (A) or multiple areas of the body (B) according to presence of inflammation (C-reactive protein > 1.0 mg/dL).

	t0			t30		
	*n*	c (%)	*p*	*n*	c (%)	*p*
Control group (*n* = 8)	8	6 (80.0)	0.447	8	7 (90.0)	0.206
Group A	7	6 (83.3)		7	7 (100.0)	
Group B	9	8 (88.9)		9	9 (100.0)	

t0 = 0 days; t30 = 30 days; *n*: number of participants in the group; c: number of cases; Chi-square association test by time according to group.

**Table 6:** Plasma concentrations of MDA, MPO and IL-1b of adult women subjected to cryolipolysis on one (group A) or multiple areas of the body (group B).

	Control Group (*n* = 8)			Group A (*n* = 7)			Group B (*n* = 9)		
	t0	t10	t30	t0	t10	t30	t0	t10	t30
MDA (Median± IQR)	2.1 ± 2.7 ^A^	3.0 ± 1.6 ^a^	4.9 ± 4.6 *	2.0 ± 1.2 ^A^	3.3 ± 1.5 *^ab^	3.2 ± 3.2 *	3.6 ± 2.5 ^B^	4.7 ± 3.7 ^b^	3.8 ± 2.6
MPO (Mean± SD)	5.9 ± 1.5 ^A^	4.9 ± 1.4 ^a^	5.2 ± 2.1^α^	4.6 ± 2.5 ^A^	7.7 ± 3.5 ^a^	9.3 ± 2.6^αβ^	9.7 ± 3.6 ^B^	14.1 ± 7.7 ^b^	11.6 ± 5.2 ^β^
IL-1β (Median± IQR)	2.9 ± 1.4 ^A^	3.6 ± 5.4	3.0 ± 3.0	4.9 ± 6.9 ^B^	4.1 ± 3.0	2.7 ± 2.4	3.2 ± 5.0 ^AB^	2.2 ± 11.7	1.8 ± 0.9

t0 = 0 days; t1 = 30 dys; Mean ± SD (Standard Deviation); Median ± IQR (Interquartile Range). * *p* < 0.05; Dunn’s test-comparing each group at times t0, t1 and t2. Different letters indicate statistical significance. Capital letters, *p* < 0.05; Dunn’s Test (MDA and IL1) or Student–Newman–Keuls test (MPO), comparing groups at t0. Lowercase letters, *p* < 0.05; Dunn’s Test (MDA e IL1) or Student–Newman–Keuls test (MPO), comparing groups at t10. Greek letters, *p* < 0.05, Dunn’s Test (MDA e IL1) or Student–Newman–Keuls test (MPO), comparing groups at t30.

## Data Availability

The datasets used during the present study are available from the corresponding author upon request.
